# Convergence of *Staphylococcus aureus* Persister and Biofilm Research: Can Biofilms Be Defined as Communities of Adherent Persister Cells?

**DOI:** 10.1371/journal.ppat.1006012

**Published:** 2016-12-29

**Authors:** Elaine M. Waters, Sarah E. Rowe, James P. O'Gara, Brian P. Conlon

**Affiliations:** 1 Department of Microbiology, School of Natural Sciences, National University of Ireland, Galway, Ireland; 2 Department of Microbiology & Immunology, University of North Carolina at Chapel Hill, Chapel Hill, North Carolina, United States of America; Nanyang Technological University, SINGAPORE

The remarkable tolerance of bacterial biofilms to antimicrobial drugs underpins their role in chronic and recurring infections. *Staphylococcus aureus* biofilms are embedded in an extracellular matrix composed of self-produced extracellular polysaccharides, DNA, and proteins or host-derived matrices such as fibrin, prompting speculation that limited drug diffusion into biofilms contributes to tolerance. However, the slow- and non-growing phenotypes of biofilm cells resemble those observed in the stationary growth phase, which is known to enrich for the highly antibiotic-tolerant persister phenotype. Indeed, recent studies have revealed that the antibiotic tolerance phenotypes of *S*. *aureus* biofilm and persister cells are strikingly similar [1–5]. Here, we will explore the idea that biofilms are enriched with adherent persister cells and that research into the biofilm and persister phenotypes has converged.

## Why Do Biofilms Exhibit High-Level Antibiotic Tolerance?

The visible extracellular polysaccharide matrix encasing many bacterial biofilms, e.g., *S*. *epidermidis* slime on infected medical devices or mucoid *Pseudomonas aeruginosa* recovered from the lungs of patients with cystic fibrosis [6, 7], has prompted the hypothesis that impaired antibiotic penetration is an important determinant in biofilm tolerance to drugs and other toxins. However, the relative contribution of impaired antibiotic penetration to biofilm tolerance remains questionable. It has been demonstrated that antibiotics do penetrate the biofilm matrix and reach the cells embedded within [8, 9] without always killing the bacteria [10]. Furthermore, cells released from biofilms are more tolerant to antibiotics than planktonic cells [11, 12], which strongly suggests that overall biofilm tolerance is not primarily the result of impaired antibiotic penetration.

Overall, it appears that the biofilm matrix does not significantly control antibiotic penetration. Rather, these data indicate that the biofilm matrix is primarily a protective agent against immune defenses during infection [13]. The altered physiology of biofilm cells reflects the unique environmental milieu and high cell density, which is likely to limit nutrient and oxygen availability and impact quorum sensing-regulated phenotypes [14]. These growth conditions appear to force a subset of biofilm cells into a stationary and persister-like state. Cells in this physiological state are primed to survive wide-ranging environmental assaults, including antibiotic challenge [2].

## How Similar Are Biofilm Cells, Stationary Phase Cells, and Persisters?

The physiology of *S*. *aureus* biofilm cells bears striking similarity to that of *S*. *aureus* persister cells. Persister cells are subpopulations of antibiotic-tolerant cells in an otherwise susceptible population. Persisters are transient phenotypic variants, which exhibit drug susceptibility upon subculture [15]. Similarly, cells that detach from antibiotic-tolerant biofilms and grow planktonically also revert to a drug-susceptible state [11, 12].

Stationary phase cultures of *S*. *aureus* demonstrate remarkable antibiotic tolerance [1, 2, 16, 17]. By definition, stationary phase cells are slow- or non-growing, a characteristic shared by biofilm cells and persister cells. Cells in such a metabolically inactive state are inherently more tolerant to antimicrobial drugs that target actively growing cells. For example, beta-lactam antibiotics are ineffective against cells that are not actively dividing and synthesizing new cell wall peptidoglycan [18]. Like biofilms, cells from stationary phase cultures also exist in a high cell density environment. At high cell densities, cells are likely to become starved of nutrients, oxygen, or both, resulting in a drop in intracellular ATP.

Recently, we reported that intracellular ATP concentration appears to be the major determinant of survival to antibiotic challenge for both stationary phase cells and persister cells in *S*. *aureus* [17]. The same may also be true for biofilm-associated cells. The limited nutrient and oxygen availability within the biofilm presumably results in reduced metabolic activity and a lower energy state, which is a hallmark of persister cells that can survive exposure to most bactericidal antibiotics.

It may be that low cell energy levels are the major determinant of antibiotic tolerance in biofilm cells, persister cells, and stationary phase cells. For example, *S*. *aureus* initiates expression of biofilm adhesins in response to a variety of external stresses, including nutrient limitation, pH stress, osmotic stress, and sublethal antibiotic challenge [6, 19, 20]. Thus, biofilm formation may also be viewed as a response by the bacteria to environmental stress that not only promotes intercellular adherence but also imposes a selective pressure for metabolically inactive, energy-depleted cells that can survive hostile growth conditions, including antibiotic challenge.

**Fig 1:**
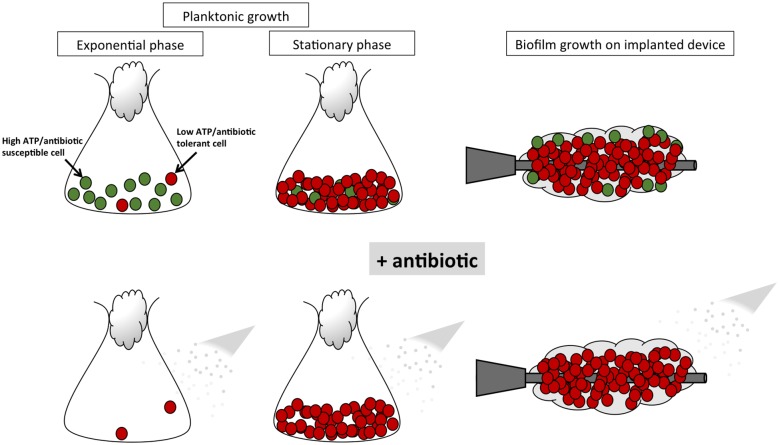
Role of persister cells in biofilm antibiotic tolerance. Antibiotic-tolerant persister cells (depicted in red) are enriched in stationary phase planktonic cultures and biofilms compared to exponential phase planktonic cultures, which are primarily composed of antibiotic-susceptible cells (depicted in green). The antibiotic susceptibility phenotype of exponential phase cells correlates with high levels of metabolic activity and ATP associated with abundant nutrients and oxygen. As cells enter stationary phase or become encased in a biofilm matrix, nutrients and oxygen are depleted and the level of intracellular ATP is reduced. Exposure to antibiotics, most of which target ATP-dependent processes, has no significant effect on metabolically inactive persister cells with low intracellular ATP levels.

However, this does not imply that all biofilm and persister cells are physiologically identical, but rather that the mechanism(s) underpinning the ability of otherwise susceptible *S*. *aureus* cells to tolerate and survive antibiotic challenge may be essentially the same (Fig 1).

## Does the Biofilm Mode of Growth Influence the Rate of Persister Cell Production in *S*. *aureus* Populations?

Exposure of planktonic staphylococcal cells to subinhibitory antibiotic concentrations induces biofilm formation [21–23]. In addition, sub-MIC (minimum inhibitory concentration) antibiotics and other external stresses enrich for drug-tolerant persister cells [15]. These observations may be important in linking the phenotypes of antibiotic tolerance and biofilm matrix production. Although antibiotics penetrate and reach high concentrations within the biofilm, penetration dynamics are influenced by the matrix [8, 10]. Together with the limited nutrient and oxygen availability, the establishment of an antibiotic concentration gradient may add an additional selective pressure for cells within the biofilm to enter a persister state upon encountering sub-MIC levels of the antibiotic, allowing them to survive the subsequent lethal concentration. Relevant to this are studies showing that the extracellular matrix deployed by *S*. *aureus* biofilms under different growth conditions, plus the age and cell density of the biofilm, significantly influences the ability of antibiotics such as rifampicin, daptomycin, vancomycin, gentamicin, and fosfomycin to kill biofilm cells [3, 4]. For instance, rifampicin can exhibit significant activity against fibrin-dependent biofilms grown in Roswell Park Memorial Institute (RPMI) media but not against FnBP/eDNA biofilms produced by the same strain in rich culture media [4]. It is therefore conceivable that the relative numbers of persister cells vary in different types of biofilm. These observations support the idea that environmental conditions (nutrient availability, oxygen concentration, cell density, and sub-MIC antibiotic levels) regulate expression of biofilm adhesins and the type of biofilm matrix produced, which influences the rate at which cells in the biofilm enter the persister state and, accordingly, the antibiotic tolerance of the biofilm.

## Can the Shared Features of Persister and Biofilm Cells Be Exploited to Better Treat Chronic Infections?

The phenotypic heterogeneity of biofilm cells, as outlined above, suggests that new treatment approaches aimed at disrupting biofilms are not necessarily going to be effective against persister cells. By contrast, new therapeutic approaches targeting persister cells should also have potential against biofilms. For example, persister cells can be killed by the acyldepsipeptide antibiotic ADEP4, which activates the nonspecific ClpP protease in an ATP-independent manner [1]. ADEP4 is active against persisters, stationary phase cells, and biofilms [1]. Similarly, the histidine kinase inhibitor NH125, which was shown to have significant antipersister activity at low concentrations, was able to kill biofilm cells and disrupt the biofilm matrix at high concentrations [24]. In *S*. *epidermidis*, the minimum bactericidal concentration of ciprofloxacin effective against biofilms can also kill most persister cells [25]. These data suggest that persister cell populations can also be used as a model to evaluate antibiofilm therapeutics. Thus, recent reports that glucose can augment daptomycin-induced killing of *S*. *aureus* persisters [26] or that the anticancer drug cisplatin [27] and cis-2-decenoic acid [28] have activity against persisters may also be indicative of antibiofilm activity.

Future research into how antibiotic-tolerant persister cells contribute to treatment failures of biofilm-associated infections can exploit advances in techniques to better study persister cells. Reporter systems developed to label persister cells could be adapted and combined with microscopy to investigate the proportion, distribution, and metabolic niche of persister cells within heterogeneous biofilm populations, as well as their response to antibiotics. Like *S*. *aureus*, the *Pseudomonas aeruginosa* biofilm phenotype is associated with antibiotic tolerance [7]. Chronic biofilm-associated infections caused by *P*. *aeruginosa* are difficult to eradicate with current antibiotic treatment regimens [7, 29], and it is feasible that such biofilms are also enriched with persister cells. Intriguingly, mannitol augments tobramycin-induced killing of *P*. *aeruginosa* persisters and biofilm cells [29] in a manner comparable to glucose-enhanced, daptomycin-induced killing of *S*. *aureus* persisters [23]. Thus, despite differences in the mechanisms of persister cell formation in gram-negative and gram-positive bacteria, the role of persister cells in biofilm tolerance may extend beyond *S*. *aureus*. Further insights into the shared antibiotic tolerance mechanisms of persister and biofilm cells are needed to direct future research into (and therapeutic options for) chronic and relapsing infections involving these important phenotypic variants of *S*. *aureus*.
